# Normal right and left ventricular volumes prospectively obtained from cardiovascular magnetic resonance in awake, healthy, 0- 12 year old children

**DOI:** 10.1186/s12968-020-0602-z

**Published:** 2020-02-03

**Authors:** Laura J. Olivieri, Jiji Jiang, Karin Hamann, Yue-Hin Loke, Adrienne Campbell-Washburn, Hui Xue, Robert McCarter, Russell Cross

**Affiliations:** 1 Division of Cardiology, Children’s National Medical Center, W3-200, 111 Michigan Ave NW, Washington, DC, 20010 USA; 2 Children’s Research Institute, Children’s National Medical Center, 111 Michigan Ave NW, Washington, DC, USA; 3 National Heart, Lung, and Blood Institute, National Institutes of Health, Bethesda, MD USA

**Keywords:** Volumetry, Pediatric cardiology, Z scores, Motion correction cine

## Abstract

**Introduction:**

Pediatric z scores are necessary to describe size and structure of the heart in growing children, however, development of an accurate z score calculator requires robust normal datasets, which are difficult to obtain with cardiovascular magnetic resonance (CMR) in children. Motion-corrected (MOCO) cines from re-binned, reconstructed real-time cine offer a free-breathing, rapid acquisition resulting in cines with high spatial and temporal resolution. In combination with child-friendly positioning and entertainment, MOCO cine technique allows for rapid cine volumetry in patients of all ages without sedation. Thus, our aim was to prospectively enroll normal infants and children birth-12 years for creation and validation of a z score calculator describing normal right ventricular (RV) and left ventricular (LV) size.

**Methods:**

With IRB approval and consent/assent, 149 normal children successfully underwent a brief noncontrast CMR on a 1.5 T scanner including MOCO cines in the short axis, and RV and LV volumes were measured. 20% of scans were re-measured for interobserver variability analyses. A general linear modeling (GLM) framework was employed to identify and properly represent the relationship between CMR-based assessments and anthropometric data. Scatter plots of model fit and Akaike’s information criteria (AIC) results were used to guide the choice among alternative models.

**Results:**

A total of 149 subjects aged 22 days–12 years (average 5.1 ± 3.6 years), with body surface area (BSA) range 0.21–1.63 m^2^ (average 0.8 ± 0.35 m^2^) were scanned. All ICC values were > 95%, reflecting excellent agreement between raters. The model that provided the best fit of volume measure to the data included BSA with higher order effects and gender as independent variables. Compared with earlier z score models, there is important additional growth inflection in early toddlerhood with similar z score prediction in later childhood.

**Conclusions:**

Free-breathing, MOCO cines allow for accurate, reliable RV and LV volumetry in a wide range of infants and children while awake. Equations predicting fit between LV and RV normal values and BSA are reported herein for purposes of creating z scores.

**Trial registration:**

clinicaltrials.gov NCT02892136, Registered 7/21/2016.

## Introduction

Cardiovascular magnetic resonance (CMR) imaging is considered the gold standard imaging method for cardiac chamber quantification, thoracic vasculature assessment and noninvasive hemodynamic assessment in children [1]. However, in growing children, a single measurement of a cardiac structure without context is minimally informative, and thus measurements are ideally interpreted in light of that child’s anthropometric data with z scores. This contextual requirement for interpreting measurements is fairly unique to pediatric medicine, and is compounded by the depth and breadth of congenital heart disease that can drastically alter cardiovascular structure and size in growing children. In this way, physicians can more clearly understand if observed measurements are within 2SD of the mean, and more importantly, if change in a measurement is expected or unexpected for growth. There are also publications in the adult cardiovascular literature touting the importance of interpreting measurements of cardiovascular structures as a percentile of mean normative values for size and age [2].

Pediatric z scores are regularly used to understand and describe normal size and structure of the heart from transthoracic echocardiography [3, 4], which enables not only the delineation of normal and abnormal, but describes the standardized magnitude of difference between an observed abnormal value and the predicted normal value. Early publications of pediatric cardiac z scores included linear measurements from 2D echo of various cardiac structures where body surface area (BSA) was the independent variable to which the measurements were related and based on [5, 6]. Others have published smaller series of niche measurements of pediatric cardiac structures, including Doppler assessments of valves, left ventricular (LV) mass [7] and coronary artery dimension [8, 9], as well as z scores for cardiac structures in the developing fetus [10]. Most recently, and most comprehensively, Lopez et al. published the most robust set of 2D echocardiography measurements that describe normal growth in children over a wide range of BSA, ages and ethnicities [11]. Z scores gauging the relative size of a cardiac structure in children are valuable to the cardiac imaging community, as well as the larger pediatric cardiology and cardiothoracic surgery community to provide standards for decision-making regarding interventions and for the evaluation of outcomes.

Prior publications exist which have sought to provide calculators for z scores of right ventricular (RV) and LV volumes in a population of children imaged with CMR. This is an inherently difficult undertaking as CMR is not typically utilized as a screening imaging modality in the way that echo is, and thus there are far fewer normal exams available. The pretest probability of presence of any cardiovascular disease is significant just by being referred for a CMR. Nevertheless, investigators have published z score calculators for various cardiovascular structures measured by CMR, notably for the thoracic aorta and pulmonary arteries [12, 13]. Some teams have been able to prospectively enroll healthy adult subjects to create a database of normal CMR measurements for adults based on age [14]. Current CMR volumetry z scores for children offer a reasonable fit based on gender and BSA [15–17], and most recently van der Ven et al. [18] published pooled analyses of pediatric volumes in 141 children based on age, but the results are difficult to apply generally due to inclusion of subjects with a wide range of age and size, as well as possible introduction of bias due to differences in anesthetic state and loading conditions in some of the subjects. Furthermore, due to paucity of normal CMR data, these z score calculators are based on data from a small number of included subjects. Sparsity of data at younger ages and smaller sizes has led to increased variability and uncertainty of estimates.

In 2016 and 2018, our group published a novel workflow for rapid, free-breathing acquisition for quantification of LV and RV volumes and systolic function, respectively, based on real-time cine with short acquisition times that were re-binned and reconstructed with high spatial and temporal resolution with motion correction (MOCO) [19, 20]. This MOCO cine approach [21] is entirely free-breathing, has optimized signal-to-noise ratio (SNR), is reconstructed inline using distributed computing in the same amount of time or less than that needed to acquire and reconstruct a typical breath-held, segmented balanced steady state free precession (bSSFP) cine stack in an awake and compliant patient [22]. Other groups have published similar successful techniques specific for rapid, free-breathing cine acquisition in children combining spiral bSSFP and compressed sensing [23]. MOCO cines, in combination with child-friendly positioning and patient entertainment systems, allows for rapid cine volumetry in patients of all ages without the use of sedation. This novel workflow provides the basis for accurate, rapid acquisition of ventricular volumes and function, and essentially allowed this current study evaluating normal ventricular sizes in children to be performed.

Our goal was to prospectively enroll normal infants and children birth-12 years for awake, free-breathing measurement of their RV and LV volumes using validated, rapid acquisition techniques for creation of a validated z score calculator describing normal RV and LV size.

## Methods

### Subject recruitment

With IRB approval and informed consent/assent where appropriate, 213 healthy children, and 5 infants with known normal intracardiac anatomy who presented for a non-sedate “feed and bundle” CMR to evaluate for a vascular ring, were prospectively enrolled in this study. Healthy subjects were recruited with flyers located at outpatient subspecialty centers located throughout the metropolitan Washington, D.C. region and via the Children’s National social media accounts. Prior to imaging, parents answered a brief questionnaire over the phone regarding the child’s past medical history and family history, so that children with significant systemic disease, genetic conditions or first degree relatives with significant heart disease in the young were excluded. Eligible families were sent a link to a preparatory movie explaining what to wear and bring to the appointment, and how to navigate the hospital quickly and efficiently, as they typically visited the hospital on evenings and weekends to increase efficiency in intake procedures and scanning. On arrival, height and weight were measured, safety screening was completed, and medical information, including a normal physical examination and absence of pediatrician concerns and emergency department visits since scheduling was again verified to ensure inclusion and exclusion criteria were met. At the conclusion of the exam, pediatricians were sent a brief clinical report, and any abnormalities were followed up with a pediatric cardiologist. Children received a gift card and small toy, and parents were reimbursed for parking/public transportation.

### CMR imaging

**Fig. 1:**
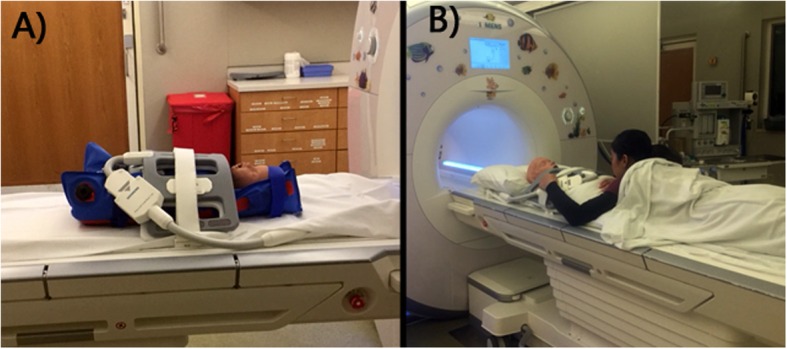
Example of positioning approach for **a** infant subjects using the “feed and bundle” approach, and **b** young children using the parents’ arms encircling them underneath the coil

CMR imaging was performed on a 1.5 T scanner (Aera, Siemens Healthineers, Erlangen Germany) with no sedation between summer 2017 – winter 2018 (18 months). Infants less than 9 months, including the 5 infants with a clinically indicated scan to evaluate for a vascular ring, were positioned and scanned using the feed and bundle technique with an infant immobilizer after feeding/burping (Fig. 1a). Children older than 9 months were offered headphones and patient entertainment system with movies for distraction. Younger children were positioned inside the magnet with their parent’s arms holding them for comfort (Fig. 1b), similar to previously published work with non-sedated imaging in young children. Older children were positioned in a typical fashion, and parents were offered a seat near the edge of the bore, if needed.

Once the children were settled, a standard clinical imaging protocol at our institution commenced using completely free-breathing techniques, as previously published by our group [19, 20], with parameters tailored to age and size. A stack of MOCO, re-binned cines was performed in the short axis projection with at least 8 slices through the ventricle in diastole to create full volumetric coverage of the ventricles. Sequence parameters were selected based on subject weight, and temporal resolution varied slightly based on subject heart rate. For example, if the heart rate is 60 bpm, the reconstructed temporal resolution will be 30 ms. For subjects in our cohort with higher heart rates, the reconstructed temporal resolution will be less than 30 ms to capture fast heart motion. (see Table 1). Phase contrast data was used to confirm the absence of significant intracardiac shunt that would alter cardiac chamber size from what was expected and prompt exclusion of that subject. To that end, flow in the ascending aorta and main pulmonary artery were performed with standard sequences (repetition time 9 ms; echo time 5 ms; matrix, 128 to 192 × 256, field of view, 250 to 350 mm; flip angle, 15; slice thickness, 5 to 7 mm; free breathing acquisition, number signal averages of 3; retrospective gating, 20 to 40 phases; in-plane and through-plane resolution optimized to patient size). Studies were assessed for quality as they were acquired, and if motion artifacts were present, sequences were repeated up to two times before the study was terminated. Studies were also checked at the time of imaging to screen for any data that would suggest a significant previously unrecognized cardiovascular abnormality, including Qp:Qs > 1.25, RV ejection fraction (RVEF) < 45%, LV ejection fraction (LVEF) < 55%. All studies took less than 20 min, and most studies took less than 10 min of scan time. See Additional files 1, 2 and 3 for examples of contoured cines for Infant, Little Kid and Big Kid cines from Table 1.

**Table 1:** Pulse sequence parameters for the motion-corrected (MOCO), re-binned cine imaging according to size

	Infant < 15 kg	Little Kid 15–35 kg	Big Kid > 35 kg
Field of view (mm)	240 × 180	280 × 210	360 × 270
Matrix	160 × 120	192 × 144	192 × 144
Slice thickness (mm)	4	6	8
Resolution (mm)	1.5 × 1.5	1.46 × 1.46	1.88 × 1.88
Flip angle (°)	50	50	50
Acceleration factor	3	4	4
Echo time (ms)	1.26	1.17	1.06
Repetition time (ms)	3.0	2.8	2.5
Views per segment	33	30	30
Reconstructed frames per cardiac cycle [22]	30	30	30

### Image analysis

**Fig. 2:**
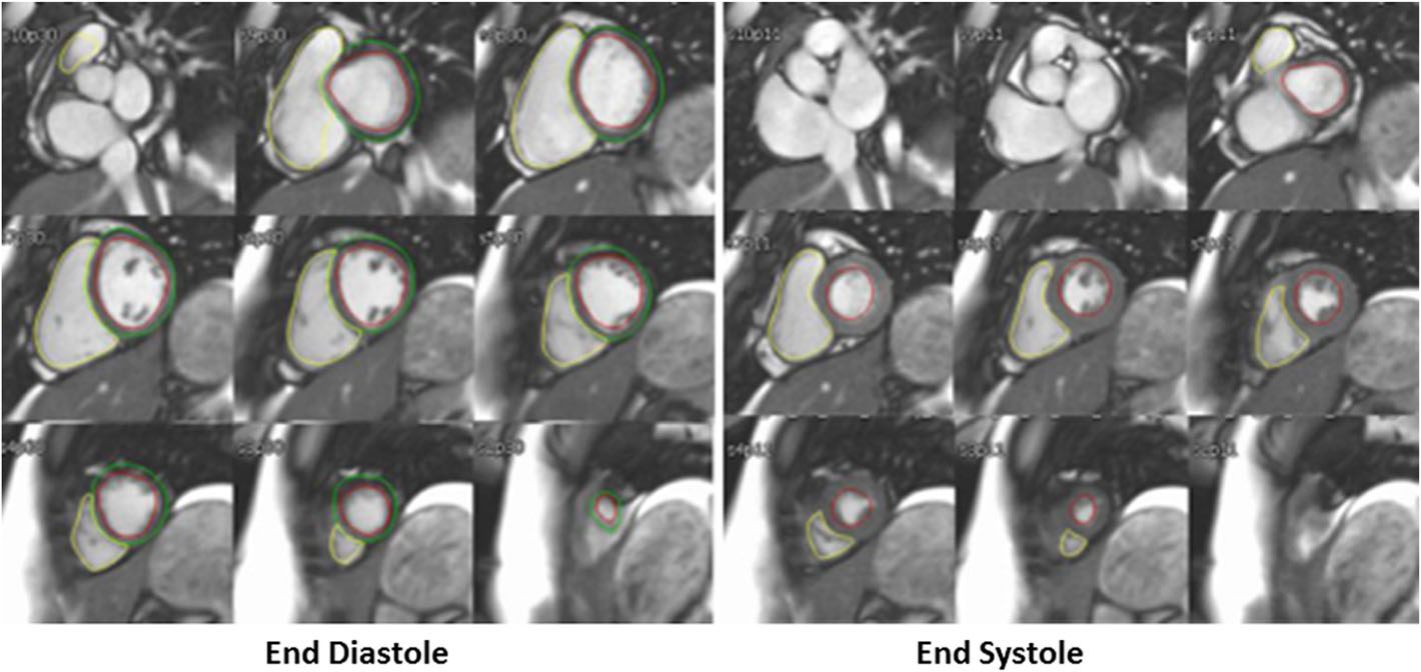
Contours of the right ventricle (RV) and left ventricle (LV) at end-diastole (left panel) and end-systole (right panel) demonstrating contouring strategy. Papillary muscle and trabeculations are included in the blood pool by tracing the blood pool-endocardial border of each ventricle in both phases

Three pediatric cardiologists with training in CMR (YL with 3 years of experience, LO with 11 years of experience and RC with 15 years of experience) supervised the studies and performed the analyses using standard offline analysis tools (MedisSuite, Medis, Leiden, the Netherlands) according to standard imaging analysis guidelines, with inclusion of papillary muscle and right ventricular trabeculations in the blood pool (Fig. 2, Additional files 1, 2 and 3) [15, 24]. Standard measurements of volumetry and flow were obtained for each study. A subset of 20% of studies were made available for a second, blinded reader from this group to perform analyses of RV and LV volumes for inter-rater reliability analyses of all 3 readers.

### Statistical methods

Analyses were implemented in STATA (*Stata Statistical Software: Release 15*. Stata Corporation, College Station, Texas, USA). A general linear modeling (GLM) framework was employed to identify and properly represent the relationship between CMR-based assessments and anthropometric data. Scatter plots for model fit and Akaike’s information criteria (AIC) results were used to guide the choice among alternative models. At each stage of model selection, model fit and normality of model residuals was evaluated to ensure that underlying assumptions were not violated. Before model development, 2-way random effects, target and rater, models were developed to estimate the intraclass correlation coefficient (ICC) reflecting the average level of agreement between paired CMR-based volumetric determinations made by each cardiologist.

## Results

**Fig. 3:**
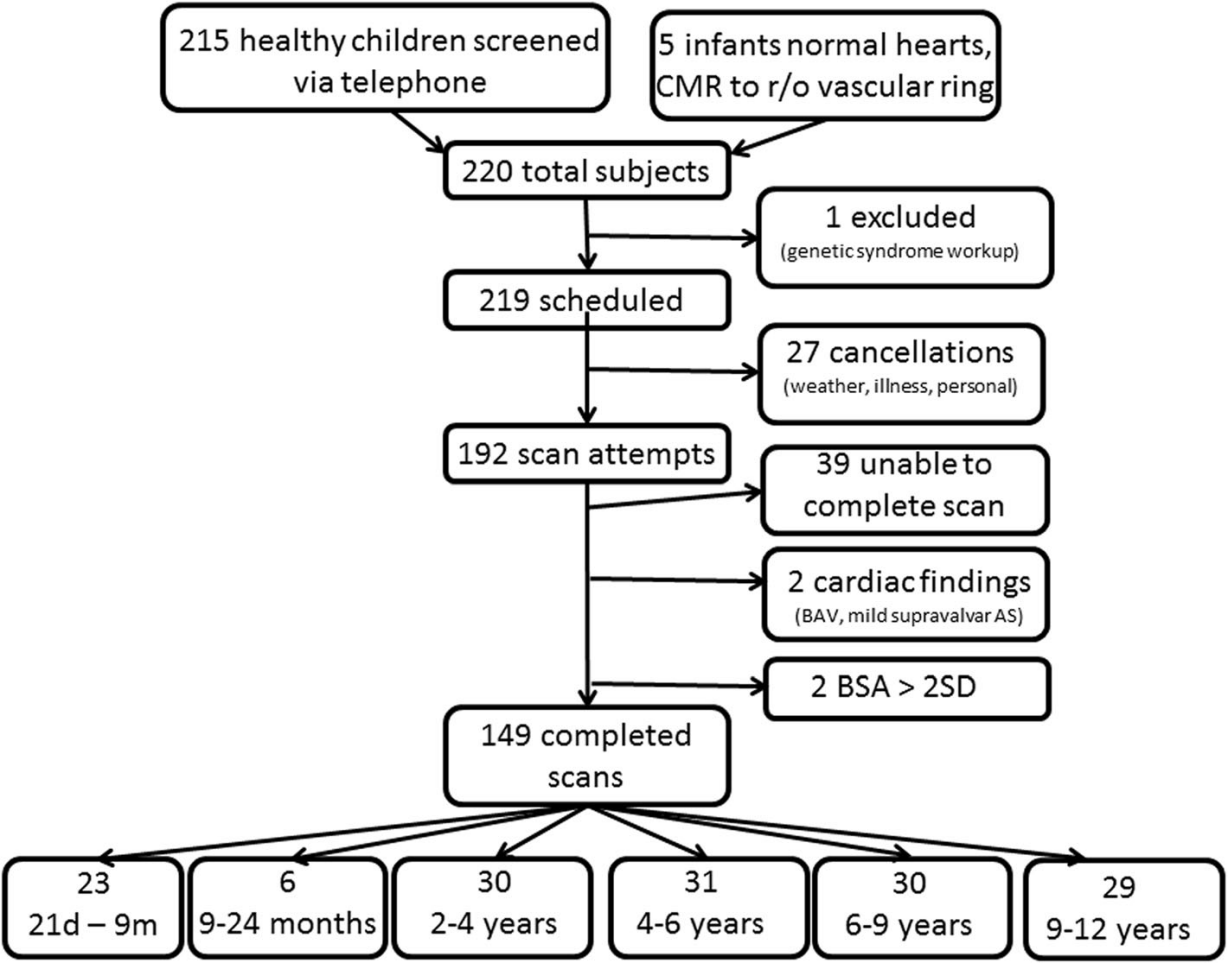
Flow diagram of enrollment for pediatric normal data project, noting exclusions where appropriate

Figure 3 illustrates the enrollment details for this study. Two hundred thirteen healthy children whose families responded to an advertisement for the study, and 5 infants with normal intracardiac anatomy (normal transthoracic echocardiogram) who were referred for a non-sedate limited CMR to evaluate for a possible vascular ring were screened for inclusion. One subject was excluded due to ongoing workup for a possible genetic abnormality. Appointments for 217 children were made for the study, and 192 subjects kept their appointment. Of these 192, 153 children (81%) were successfully scanned without sedation. Two children were excluded from the final analysis due to the presence of previously unknown cardiovascular findings found at the time of the CMR (bicuspid aortic valve in a 6 year old boy without significant family history, and mild supravalar aortic stenosis in a 2 year old girl). Thirty-nine children were unable to complete the full scan due to age-appropriate anxiety/behavior. Two additional children had BSA’s that exceeded 2SD of the normal range, and so their data were also excluded. A final total of 149 children were included in the study.

Demographics of this successfully imaged normal cohort of 149 children are shown in Table 2. Subjects ranged in age 22 days − 12 years (average 5.1 ± 3.6 years), with BSA range0.21–1.63 m^2^ (average 0.8 ± 0.35 m^2^), and heart rate range 51–197 bpm, average 100 ± 25 bpm.

**Table 2:** Demographics of prospectively enrolled normal pediatric cohort

Age	5.1 ± 3.6 years (21 days to 12 years)
Height	108 ± 30 cm (43–168)
Weight	22.3 ± 13.7 kg (3.8–69.2)
BSA	0.8 ± − 0.4 m^2^ (0.21–1.63)
Gender	53% Female
Race	73%Caucasian
	13% Black
	3% Asian/Pacific Islander
	9% More than 1 race
	2% Other
Heart Rate	100 ± 25 bpm (51–197)
RV ejection fraction	62 ± 6% (45–78%)
LV ejection fraction	62 ± 4% (55–74%)
Qp:Qs	1.04 ± 0.08 (0.80–1.25)

*BSA* body surface area, *LV* left ventricular, *Qp:Qs* pulmonary to systemic flow, *RV* right ventricualr

### Interobserver analysis

**Fig. 4:**
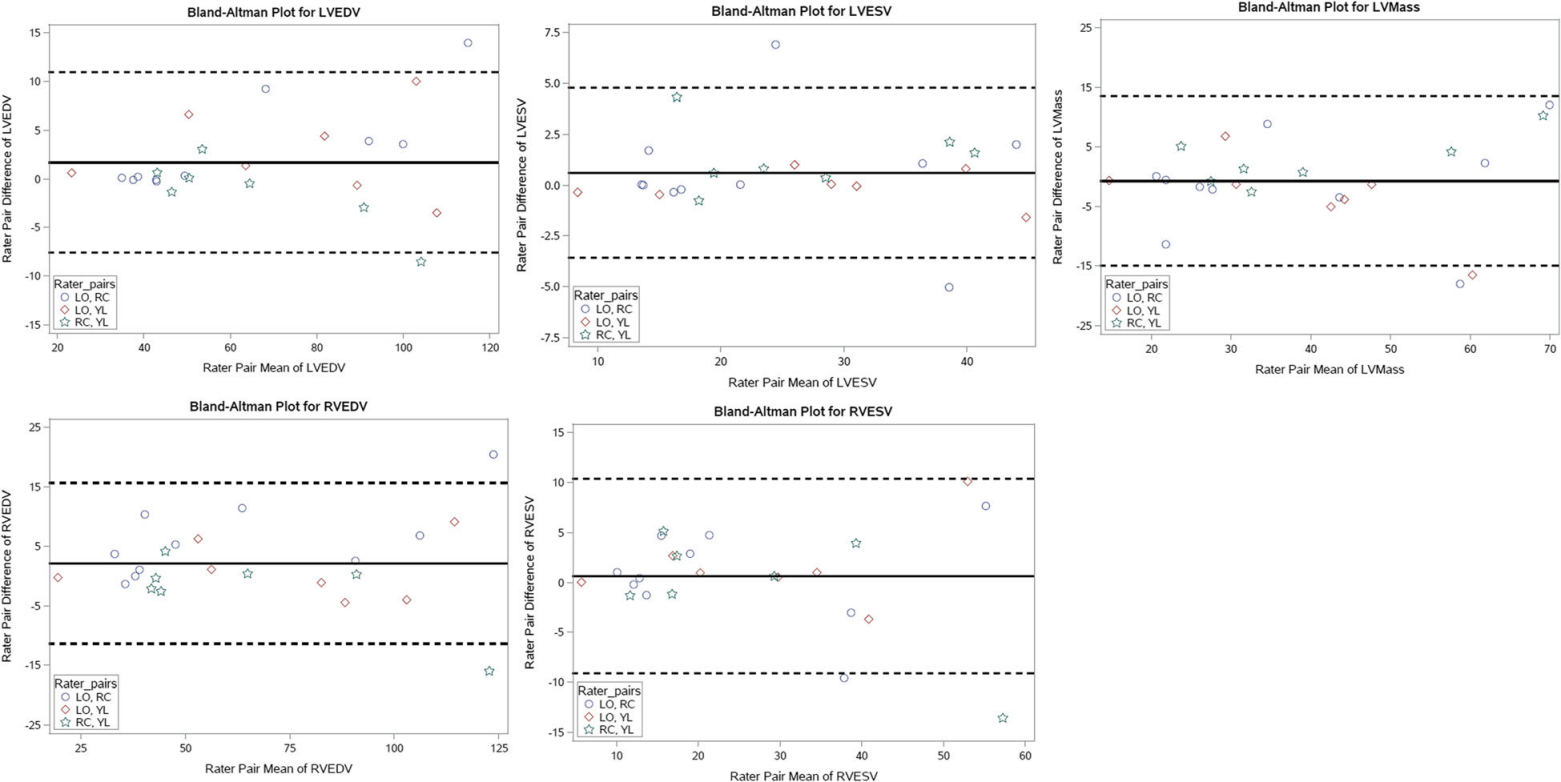
Bland-Altman analyses for each of the 5 measured endpoints (LV end-diastolic volume (LVEDV), LV end-systolic volume (LVESV), LV mass, RV end-diastolic volume (RVEDV), RV end-systolic volume (RVESV) for each of the 3 observers (LO, RC, YL)

ICC estimates reflecting the average level of agreement between all possible pairs of three cardiologists’ CMR-based volumetric determinations were obtained, and all ICC values were above 95%, reflecting excellent agreement across each of the pairs of raters on each volumetric determination. Detailed results for the ICC analyses are found in Table 3, and depicted by Bland-Altman analysis in Fig. 4.

**Table 3:** Intraclass correlation coefficients for each measured variable between each of the 3 CMR readers. Values greater than 0.9 reflect excellent agreement. Inter-rater agreement is very strong

Outcome	Rater Pair	Intraclass Correlation Results	
		Average ICC	95% Conf. Interval
LVEDV	LO-RC	0.993	(0.973, 0.998)
	LO-YL	0.994	(0.966, 0.999)
	RC-YL	0.994	(0.965, 0.999)
LVESV	LO-RC	0.984	(0.935, 0.996)
	LO-YL	0.999	(0.993, 0.999)
	RC-YL	0.993	(0.960, 0.999)
LVSV	LO-RC	0.985	(0.941, 0.996)
	LO-YL	0.986	(0.921, 0.998)
	RC-YL	0.979	(0.880, 0.996)
LV Mass	LO-RC	0.945	(0.779, 0.986)
	LO-YL	0.944	(0.673, 0.990)
	RC-YL	0.984	(0.906, 0.997)
RVEDV	LO-RC	0.990	(0.960, 0.998)
	LO-YL	0.994	(0.965, 0.999)
	RC-YL	0.989	(0.938, 0.998)
RVESV	LO-RC	0.974	(0.896, 0.994)
	LO-YL	0.983	(0.898, 0.997)
	RC-YL	0.964	(0.790, 0.994)
RVSV	LO-RC	0.982	(0.929, 0.996)
	LO-YL	0.995	(0.969, 0.999)
	RC-YL	0.994	(0.965, 0.999)

*LVEDV* left ventricular end-diastolic volume, *LVESV* left ventricular end-systolic volume, *LVSV* left ventricular stroke volume, *RVEDV* right ventricular end-diastolic volume, *RVESV* right ventricular end-systolic volume, *RVSV* right ventricular stroke volume

### Model fitting

Using the procedures described in methods, the final best fitting model to estimate normative values for each of the CMR-based volumetric parameters focused on BSA as the key metric:

$$ Outcome=\beta 0+\beta 1\ast BSA+\beta 2\ast BSA\hat{\mkern6mu} 2+\beta 3\ast sex $$

**Fig. 5:**
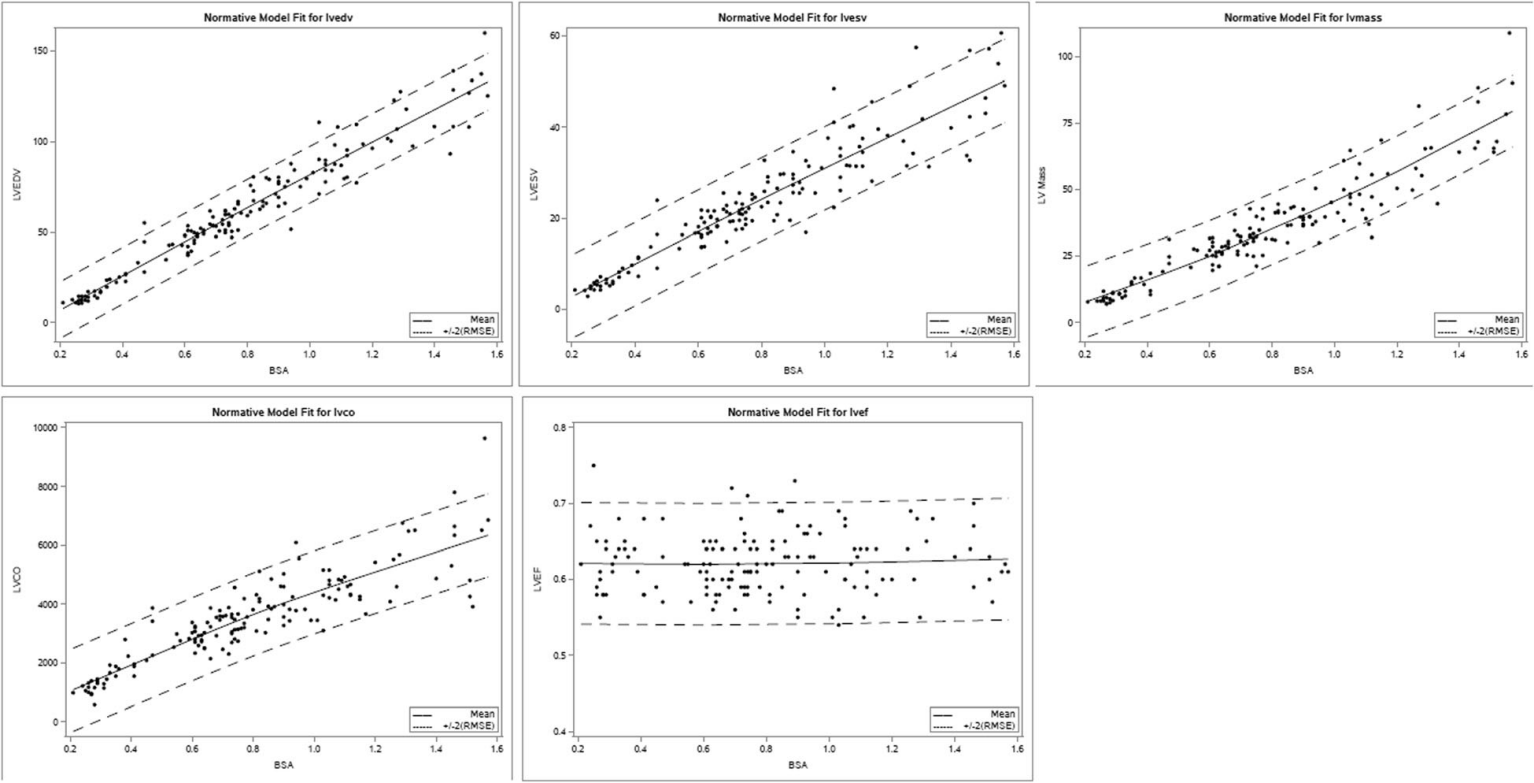
Scatterplots of LVEDV, LVESV, LV mass, LVEF and LV cardiac output (LVCO) with sample best fit lines, and Z = + 2, Z = -2 lines for the enrolled pediatric normal cohort

**Fig. 6:**
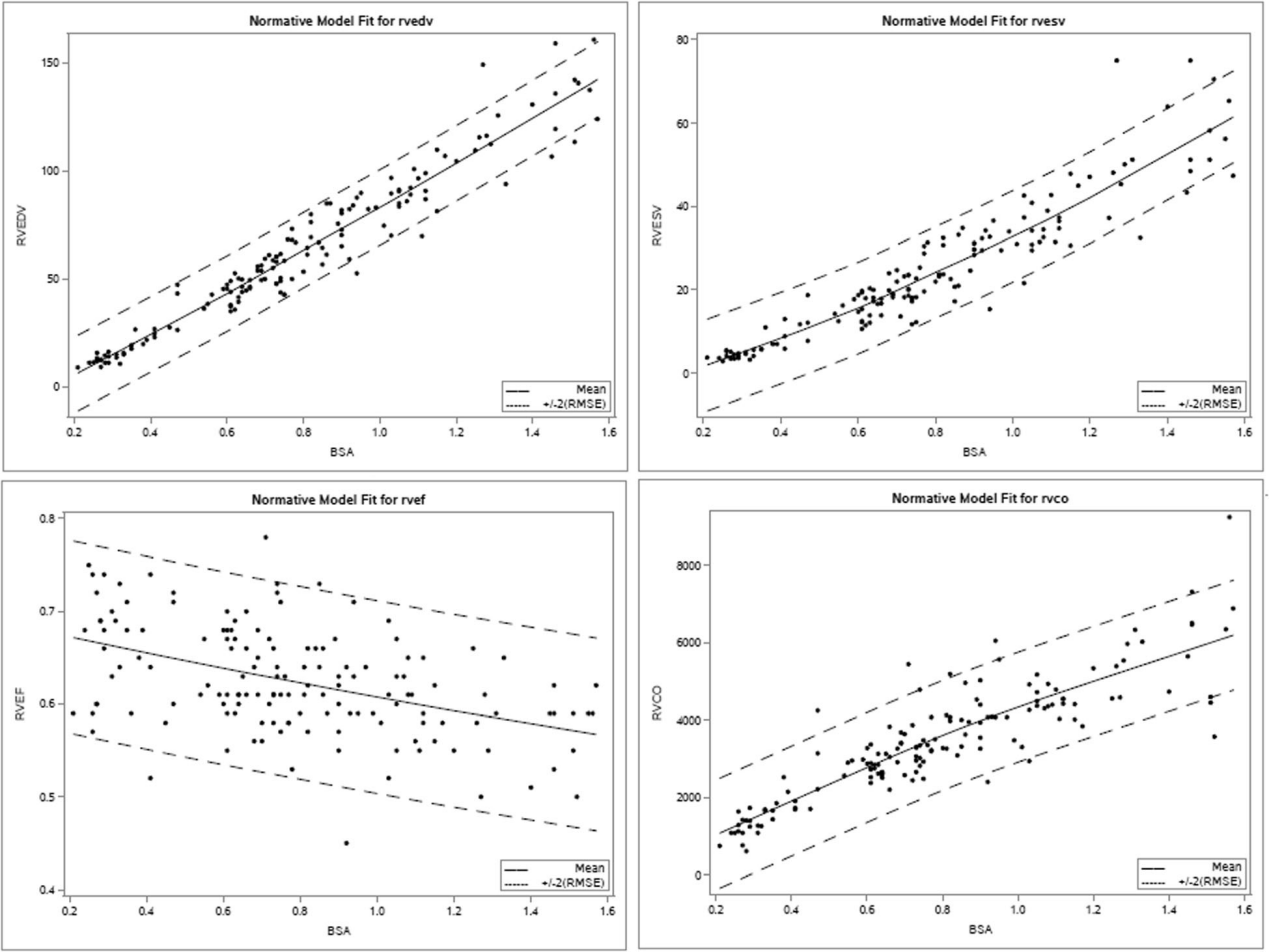
Scatterplots of RVEDV, RVESV, RVEF and RV cardiac output (RVCO) with sample best fit lines, and Z = + 2, Z = -2 lines for the enrolled pediatric normal cohort

We evaluated the alternative transformations, including identity log, square root and each created acceptably normalized vol. However, the model that provided the best fit to the data included untransformed volume measurements. This model provided an excellent fit to the normative data with normally distributed residuals and it explained more than 90% of the variance of all volumetric outcomes except LV cardiac output and RV cardiac output, for which it explained more than 78–79%% of the variance. Before choosing this model, models with more parameters, including heart rate during CMR, weight and height instead of BSA, age, as well as additional higher order effects were evaluated. Models including age in addition to BSA were considered but were abandoned due to concern for over-fitting and variance inflation because of the high correlation between BSA and age (*r* > 0.9). None of the alternative models excluding age offered a better fit by visual inspection and confirmed by AIC. In spite of the lack of a statistically significant relationship between RV ejection fraction (RVEF) and LV ejection fraction (LVEF) and BSA in this dataset, all model parameters needed for the z score determinations are included in Table 4, and plots depicting final model fits are found in Figs. 5 and 6 (left heart and right heart, respectively), with Z = + 2, 0, − 2 lines.

**Table 4:** Final regression analysis using model for calculation of z score, Outcome = β0 + β1*BSA + β2*BSA^2 + β3*sex

Measurement	R^2	RMSE	β0	β1	β2	β3
LVEDV	0.943	7.833	−15.03043	98.49337	−4.022686	5.115424
LVESV	0.872	4.591	−5.676169	37.48608	−1.767246	2.037896
LVSV	0.943	4.892	−9.354262	61.00728	−2.255442	3.077528
LVCO	0.786	705.779	−54.81287	5073.386	− 697.3501	173.8217
LV Mass	0.878	6.715	−1.310437	36.1225	9.161217	2.926009
RVEDV	0.94	8.736	−14.83598	89.43943	5.66115	5.666917
RVESV	0.882	5.496	−5.44392	28.04597	8.750498	2.760762
RVSV	0.927	5.464	−9.392059	61.39346	−3.089348	2.906154
RVCO	0.776	710.172	−59.11051	5082.184	− 756.974	172.5073

*Note: Sex is coded 1 for male, 0 for female, thus β3 is added for males and is omitted for females

A z-score calculator is provided to reflect the deviation of patient’s measured volume from the norm estimated from the model using the following computation:

$$ Z- score\ (vol)= Patient\  Vol- Model\ Estimate/ RMSE $$

### Comparison with prior z score calculators

**Fig. 7:**
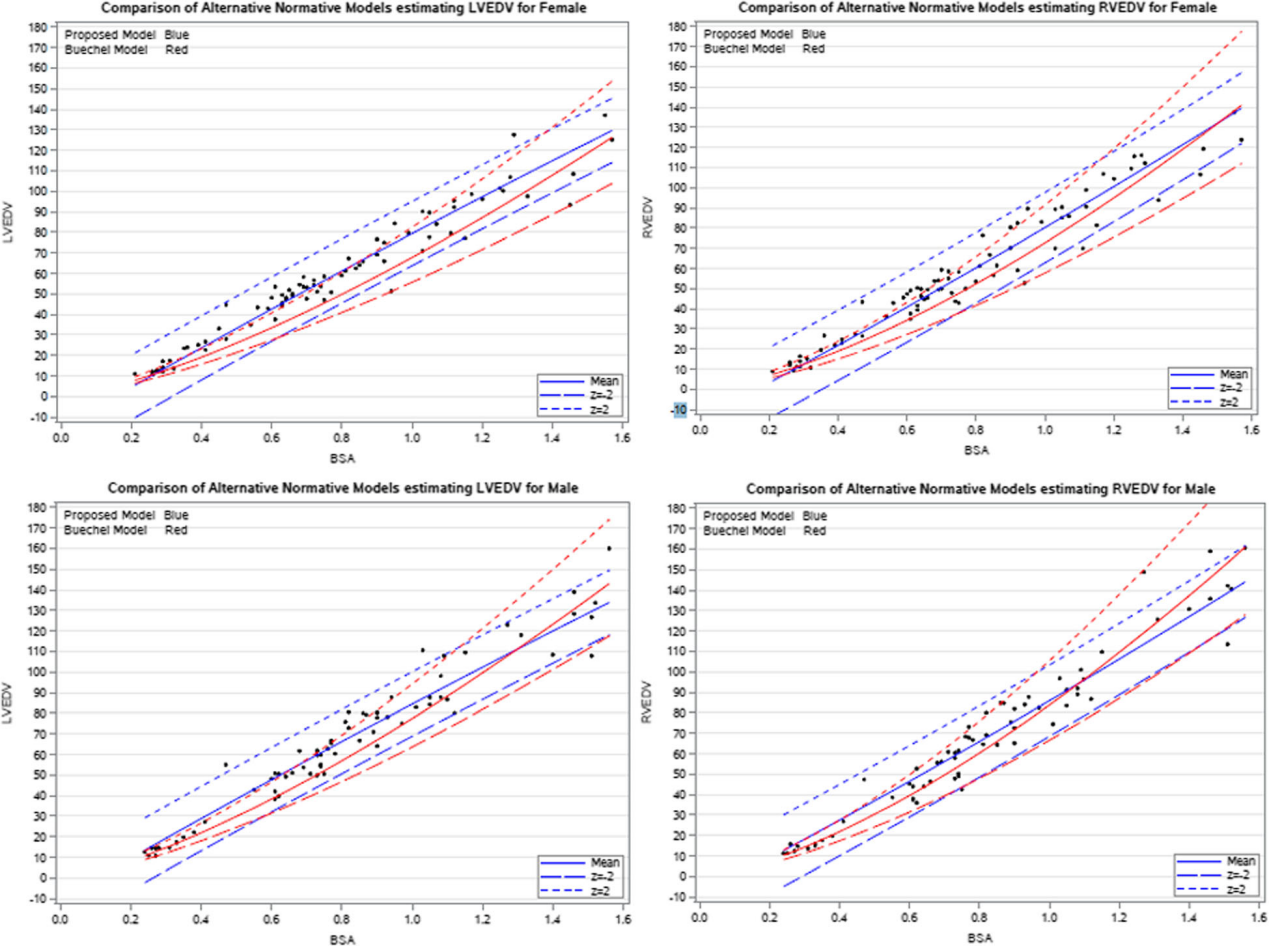
Graph depicting the relationship between the previously published Buechel z score equations (mean, +2SD, − 2SD) and our z score equations (mean, +2SD, − 2SD) for LVEDV vs. body surface area (BSA) and RVEDV vs. BSA

We sought to compare our z score equations to previously derived z score equations in use by other CMR labs, although they have limitations. Figure 7 shows the proposed z score model best fit, z = 2, and z = − 2 for RV end-diastolic volume (RVEDV) and LV end-diastolic volume (LVEDV) superimposed on the same curves using the Buechel model. There is important additional growth inflection in early toddlerhood in our model which is not present in the Buechel model, however both models generally predict similar z scores for a given volume measurement.

## Discussion

This study provides and evaluates a set of z score calculators for normal RV and LV sizes based on robust, validated measurements from 149 healthy, awake and free-breathing children 0–12 years old. This is a significant contribution to the field, made possible by the novel MOCO cine workflow enabling very rapid acquisition of cines for volumetry in children without sedation, so that all children are scanned prospectively with identical imaging methods and identical loading conditions (i.e. no sedation). Because somatic growth and cardiovascular growth are not linearly related, this dataset is highly necessary and, until now, has not been feasible due to requirements for breath-holds and long acquisition times. Some authors have created more comprehensive sets of z scores, such as Campens et al. who published an aortic dimension z score calculator for ages 0–85 [25]. This is well-meaning but falls short of the goal of creating a model for a more specific age range, because somatic growth velocities (measured by height, weight, BSA) differ significantly from birth to 12 years of age vs. age 12 and up. Creating a unifying model of cardiac growth and accompanying z score calculator for cardiac volumes for children 0–12 years was our specific goal because other studies describe cardiac chamber size very well for those 12 years of age and up [16, 17], and somatic growth is incredibly different in children age 12 and up, which makes creation of one unifying model for birth-18 a difficult if not impossible task. In contrast, some authors have created excellent and well-fitted models for very specific circumstances that may not be as useful to the general field, such as Cantinotti et al. who report a set of z score calculators for cardiac structures from a group of Caucasian infants and toddlers measured with echo [26], or Choudhry et al. who rigorously created a z score calculator for M-mode measurements of the LV in infants with weight 500–2000 g [27].

Most recently, van der Ven et al. [18] pooled previously obtained data in a multi-center group of high-volume pediatric programs to produce volumetry analyses in 141 healthy children 0–18 years old, using segmented bSSFP cine imaging. This is an important contribution, however limitations include ethnicity of the population (100% Caucasian), and underrepresentation of younger children, with 8% (12/141) under 7 years of age, compared to our prospectively enrolled population where 61% (91/149) were less than 7 years of age. Additionally, somewhat heterogeneous imaging parameters and loading conditions were noted throughout the study, with some children sedated for an otherwise clinically indicated musculoskeletal MRI, some had free-breathing, averaged, segmented cine imaging, and some had breath-held segmented bSSFP imaging. Finally, van der Ven et al’s analyses also excluded papillary muscles and trabeculations from the blood pool, our and many other centers’ standard clinical practice, which is a factor when comparing values between the two studies [24, 28]. Each method has cited literature supporting its application [29, 30], thus it is important for the imager to understand which study to use for accurate z score generation based on their contouring method [28]. Generally speaking, our model differs from the Lopez et al. Pediatric Heart Network echo paper [11], in that we found a linear relationship between BSA and cardiac chamber size, whereas they developed a model based upon a ratio estimator indexed to BSA raised to a fitted power. In addition, we found a constant sex difference, indicating modestly larger chamber sizes in males across all age groups.

Our group’s decision to use awake, free-breathing acquisitions using the real-time, re-binning technique was deliberate, acknowledging that there are both advantages and disadvantages to this decision. Advantages of this technique are that it allows for rapid acquisition of volumetry data in all age groups, including babies and young children, without the need for sedation or mechanical ventilation breath-holds. Our prior work has demonstrated that images can be reliably obtained in a fraction of the time needed for breath-holding, and yield volumetric measurements that are clinically comparable to traditional breath-held techniques [19, 20]. It has been shown that breath-holding techniques in fact alter normal physiology [31] and can result in significantly different measurement results when compared to results based on free-breathing. Because of this, it has been suggested that free-breathing techniques are likely a better measures of true physiology. An admitted disadvantage of the free-breathing technique that was chosen is that most currently available clinical criteria and patient care guidelines are based on data acquired using traditional breath-hold approaches. While this understandably raises questions regarding the appropriateness of applying these guidelines using free-breathing data, it is our belief that future trends in CMR imaging will be moving in the direction of free-breathing techniques as acquisition and reconstruction technology continues to advance, obviating the need for breath-hold imaging. This will be particularly important in the younger age pediatric population.

Some have criticized the use of BSA as the independent variable in a z score calculator as z scores can be overestimated with very elevated BSA’s; and these authors have advocated for use of lean or ideal body mass instead of calculated body surface area [32, 33]. Creating a cohort of “normal” imaging studies for derivation of z scores is difficult. For cost and utilization reasons, a retrospective sample of convenience is typically used for calculation of z scores, which requires fastidious attention to the clinical details of that patient’s encounter that led to the imaging to avoid inadvertent inclusion of abnormal studies. In addition, patients should be of normal size (i.e. height, weight and BSA between − 2 and + 2 SD according to the Centers for Disease Control and Prevention (CDC) growth curves) to avoid skewing the relationship between cardiac structures and somatic growth [34]. Finally, since various genetic syndromes predictably affect somatic growth, it is appropriate to develop separate, condition-specific z scores for structures of interest, for example in interpreting linear dimension of girls and women with Turner syndrome, who are known to be at risk for aortic dilation, dissection and rupture, but who have small BSA compared to the general population [35].

There are limitations to this work, most notably the difficulty in successfully scanning children between 9 and 24 months, with the implication that the size of cardiac chambers may be mis-represented in this age group. Our group recruited heavily in the 0–9 months and 2–4 years categories, representing the last 6 months of the study period, in order to minimize the impact of this lack of toddler data, reducing the likelihood that volume estimates would be mis-estimated. In addition, the study is limited by the fact that re-binned MOCO imaging was used, reconstructed on a gadgetron framework, with subjects that have higher heart rates on average than the average heart rate of the cohorts included in the numerous studies which have found no difference between volumetry from these sequences and segmented free breathing or breath hold bSSFP cine imaging.

## Conclusion

In conclusion, free-breathing, MOCO cines re-binned and reconstructed allow for accurate, reliable RV and LV volumetry in a wide range of infants and children while awake. Equations predicting fit between RV and LV normal values and BSA are reported here for purposes of creating z scores.

## Supplementary information

**Additional file 1.** Example cine of an infant from the study using the Infant imaging parameters and the MOCO acquisition.

**Additional file 2.** Example cine of a 4 year old child from the study using the Little Kid imaging parameters and the MOCO acquisition.

**Additional file 3.** Example cine of a 9 year old child from the study using the Bid Kid imaging parameters and the MOCO acquisition.

## Data Availability

The datasets generated and/or analysed during the current study are not publicly available due to ongoing analyses with these datasets. They could be made available from the corresponding author on reasonable request.

## Abbreviations

AIC: Akaike’s information criteria
BSA: Body surface area
bSSFP: Balanced steady state free precession
CMR: Cardiovascular magnetic resonance
GLM: General linear modeling
ICC: Intraclass correlation coefficient
LV: Left ventricle/left ventricular
LVCO: Left ventricular cardiac output
LVEDV: Left ventricular end-diastolic volume
LVEF: Left ventricular ejection fraction
LVESV: Left ventricular end-systolic volume
LVSV: Left ventricular stroke volume
MOCO: Motion corrected
RV: Right ventricle/right ventricular
RVCO: Right ventricular cardiac output
RVEDV: Right ventricular end-diastolic volume
RVEF: Right ventricular ejection fraction
RVEF: Right ventricular ejection fraction
RVESV: Right ventricular end-systolic volume
RVSV: Right ventricular stroke volume
SNR: Signal-to-noise ratio

## References

[1] Fratz S, Chung T, Greil GF, Samyn MM, Taylor AM, Valsangiacomo Buechel ER (2013). Guidelines and protocols for cardiovascular magnetic resonance in children and adults with congenital heart disease: SCMR expert consensus group on congenital heart disease. J Cardiovasc Magn Reson.

[2] Pfaffenberger S, Bartko P, Graf A, Pernicka E, Babayev J, Lolic E (2013). Size matters! Impact of age, sex, height, and weight on the normal heart size. Circ Cardiovasc Imaging.

[3] Daubeney PE, Blackstone EH, Weintraub RG, Slavik Z, Scanlon J, Webber SA (1999). Relationship of the dimension of cardiac structures to body size: an echocardiographic study in normal infants and children. Cardiol Young.

[4] Roman MJ, Devereux RB, Kramer-Fox R, O’Loughlin J (1989). Two-dimensional echocardiographic aortic root dimensions in normal children and adults. Am J Cardiol.

[5] Pettersen MD, Du W, Skeens ME, Humes RA (2008). Regression equations for calculation of z scores of cardiac structures in a large cohort of healthy infants, children, and adolescents: an echocardiographic study. J Am Soc Echocardiogr.

[6] Colan S, Cohen M, Lai W, Geva T, Mertens L (2009). Normal echocardiographic values for cardiovascular structures. Echocardiogr Pediatr Congenit Heart Dis.

[7] Foster BJ, Mackie AS, Mitsnefes M, Ali H, Mamber S, Colan SD (2008). A novel method of expressing left ventricular mass relative to body size in children. Circulation.

[8] Kobayashi T, Fuse S, Sakamoto N, Mikami M, Ogawa S, Hamaoka K (2016). A New Z Score Curve of the Coronary Arterial Internal Diameter Using the Lambda-Mu-Sigma Method in a Pediatric Population. J Am Soc Echocardiogr.

[9] Olivieri L, Arling B, Friberg M, Sable C (2009). Coronary artery Z score regression equations and calculators derived from a large heterogeneous population of children undergoing echocardiography. J Am Soc Echocardiogr.

[10] Krishnan A, Pike JI, McCarter R, Fulgium AL, Wilson E, Donofrio MT (2016). Predictive models for Normal fetal cardiac structures. J Am Soc Echocardiogr.

[11] Lopez L, Colan S, Stylianou M, et al. Relationship of Echocardiographic Z Scores Adjusted for Body Surface Area to Age, Sex, Race, and Ethnicity: The Pediatric Heart Network Normal Echocardiogram Database. Circ Cardiovasc Imaging. 2017;10(11):e006979. 10.1161/CIRCIMAGING.117.006979 10.1161/CIRCIMAGING.117.006979 PMC5812349 29138232

[12] Knobel Z, Kellenberger CJ, Kaiser T, Albisetti M, Bergsträsser E, Buechel ERV (2011). Geometry and dimensions of the pulmonary artery bifurcation in children and adolescents: assessment in vivo by contrast-enhanced MR-angiography. Int J Card Imaging.

[13] Kaiser T, Kellenberger CJ, Albisetti M, Bergsträsser E, Valsangiacomo Buechel ER (2008). Normal values for aortic diameters in children and adolescents--assessment in vivo by contrast-enhanced CMR-angiography. J Cardiovasc Magn Reson.

[14] Burman ED, Keegan J, Kilner PJ (2016). Pulmonary artery diameters, cross sectional areas and area changes measured by cine cardiovascular magnetic resonance in healthy volunteers. J Cardiovasc Magn Reson.

[15] Buechel EV, Kaiser T, Jackson C, Schmitz A, Kellenberger CJ (2009). Normal right- and left ventricular volumes and myocardial mass in children measured by steady state free precession cardiovascular magnetic resonance. J Cardiovasc Magn Reson.

[16] Kawel-Boehm N, Maceira A, Valsangiacomo-Buechel ER, Vogel-Claussen J, Turkbey EB, Williams R (2015). Normal values for cardiovascular magnetic resonance in adults and children. J Cardiovasc Magn Reson.

[17] Sarikouch S, Peters B, Gutberlet M, Leismann B, Kelter-Kloepping A, Koerperich H (2010). Sex-specific pediatric percentiles for ventricular size and mass as reference values for cardiac MRI: assessment by steady-state free-precession and phase-contrast MRI flow. Circ Cardiovasc Imaging.

[18] van der Ven JPG, Sadighy Z, Valsangiacomo Buechel ER, Sarikouch S, Robbers-Visser D, Kellenberger CJ, et al. Multicentre reference values for cardiac magnetic resonance imaging derived ventricular size and function for children aged 0–18 years. Eur Heart J Cardiovasc Imaging. 2019; Available from: https://academic.oup.com/ehjcimaging/advance-article/doi/10.1093/ehjci/jez164/5529184. Cited 2019 Sep 26. 10.1093/ehjci/jez164 PMC6923680 31280290

[19] Cross R, Olivieri L, O’Brien K, Kellman P, Xue H, Hansen M (2016). Improved workflow for quantification of left ventricular volumes and mass using free-breathing motion corrected cine imaging. J Cardiovasc Magn Reson.

[20] Merlocco A, Olivieri L, Kellman P, Xue H, Cross R. Improved Workflow for Quantification of Right Ventricular Volumes Using Free-Breathing Motion Corrected Cine Imaging. Pediatr Cardiol. 2019;40(1):79–88. 10.1007/s00246-018-1963-z 10.1007/s00246-018-1963-z PMC9581608 30136135

[21] Xue H, Kellman P, Larocca G, Arai AE, Hansen MS (2013). High spatial and temporal resolution retrospective cine cardiovascular magnetic resonance from shortened free breathing real-time acquisitions. J Cardiovasc Magn Reson.

[22] Xue H, Inati S, Sørensen TS, Kellman P, Hansen MS. Distributed MRI reconstruction using gadgetron-based cloud computing. Magn Reson Med. 2014. 10.1002/mrm.25213 PMC6328377 24687458

[23] Steeden JA, Kowalik GT, Tann O, Hughes M, Mortensen KH, Muthurangu V (2018). Real-time assessment of right and left ventricular volumes and function in children using high spatiotemporal resolution spiral bSSFP with compressed sensing. J Cardiovasc Magn Reson.

[24] Papavassiliu T, Kühl HP, Schröder M, Süselbeck T, Bondarenko O, Böhm CK (2005). Effect of Endocardial Trabeculae on left ventricular measurements and measurement reproducibility at cardiovascular MR imaging. Radiology.

[25] Campens L, Demulier L, De Groote K, Vandekerckhove K, De Wolf D, Roman MJ (2014). Reference values for echocardiographic assessment of the diameter of the aortic root and ascending aorta spanning all age categories. Am J Cardiol.

[26] Cantinotti M, Scalese M, Murzi B, Assanta N, Spadoni I, Festa P (2014). Echocardiographic nomograms for ventricular, valvular and arterial dimensions in caucasian children with a special focus on neonates, infants and toddlers. J Am Soc Echocardiogr.

[27] Choudhry S, Salter A, Cunningham TW, Levy PT, Nguyen HH, Wallendorf M (2017). Normative Left Ventricular M-Mode Echocardiographic Values in Preterm Infants up to 2 kg. J Am Soc Echocardiogr.

[28] Schulz-Menger J, Bluemke DA, Bremerich J, Flamm SD, Fogel MA, Friedrich MG, et al. Standardized image interpretation and post processing in cardiovascular magnetic resonance: Society for Cardiovascular Magnetic Resonance (SCMR) Board of Trustees Task Force on Standardized Post Processing. J Cardiovasc Magn Reson. 2013;15 Available from: https://jcmr-online.biomedcentral.com/articles/10.1186/1532-429X-15-35. Cited 2019 Sep 26. 10.1186/1532-429X-15-35 PMC3695769 23634753

[29] Natori S, Lai S, Finn JP, Gomes AS, Hundley WG, Jerosch-Herold M (2006). Cardiovascular function in multi-ethnic study of atherosclerosis: Normal values by age, sex, and ethnicity. Am J Roentgenol.

[30] Hudsmith L, Petersen S, Francis J, Robson M, Neubauer S (2005). Normal Human Left and Right Ventricular and Left Atrial Dimensions Using Steady State Free Precession Magnetic Resonance Imaging. J Cardiovasc Magn Reson.

[31] Claessen G, Claus P, Delcroix M, Bogaert J, La Gerche A, Heidbuchel H (2014). Interaction between respiration and right versus left ventricular volumes at rest and during exercise: a real-time cardiac magnetic resonance study. Am J Physiol Heart Circ Physiol.

[32] Neilan TG, Pradhan AD, King ME, Weyman AE (2009). Derivation of a size-independent variable for scaling of cardiac dimensions in a normal paediatric population. Eur J Echocardiogr.

[33] Foster BJ, Khoury PR, Kimball TR, Mackie AS, Mitsnefes M (2016). New Reference Centiles for Left Ventricular Mass Relative to Lean Body Mass in Children. J Am Soc Echocardiogr.

[34] Cantinotti M, Scalese M, Molinaro S, Murzi B, Passino C (2012). Limitations of current echocardiographic nomograms for left ventricular, valvular, and arterial dimensions in children: a critical review. J Am Soc Echocardiogr.

[35] Quezada E, Lapidus J, Shaughnessy R, Chen Z, Silberbach M (2015). Aortic dimensions in turner syndrome. Am J Med Genet A.

